# Restoratively driven planning for implants in the posterior maxilla - Part 2: implant planning, biomechanics and prosthodontic planning a proposed prosthodontic complexity index

**DOI:** 10.1038/s41415-023-6440-2

**Published:** 2023-11-10

**Authors:** Elizabeth M. King, Jonathon Schofield

**Affiliations:** 41415450031001 Consultant Senior Lecturer in Restorative Dentistry, University of Bristol, Bristol Dental School, UK; 41415450031002 Senior Clinical Lecturer, University of Bristol, Bristol Dental School, UK

## Abstract

Restoratively driven implant planning in the posterior maxilla requires a comprehensive understanding of the anatomical and physiological changes of the alveolar bone following tooth extraction and sinus augmentation. As a part of restoratively driven planning, alveolar bone, inter-arch relationships, proposed crown-implant ratio and anticipated non-axial loading should be assessed pre-operatively. This helps determine the prosthodontic and surgical aspects of implant treatment, such as prosthesis design, implant number, implant angulation, implant length and the necessity for additional bone grafting procedures. However, currently no implant planning classification is restoratively driven and include these important prosthodontic considerations. Therefore, a new index - the Posterior Maxilla Prosthodontic Index - is defined to encourage restoratively driven implant planning in the posterior maxilla.

## Key points

- This review describes the clinical aspects that influence prosthodontic planning in the atrophic posterior maxilla.
- Assessment of anatomical and biomechanical factors affecting prosthesis and implant design in the posterior maxilla are discussed.
- The Posterior Maxilla Prosthodontic Index proposed in this article identifies the prosthodontic aspects of clinical examination and treatment planning required for restoratively driven implant planning in the posterior maxilla.

## Introduction

Restoratively driven planning for implants in the posterior maxilla presents unique challenges compared to other edentulous sites, as described in the first article of this series.^1^ Comprehensive assessment of multiple clinical variables, including the implant site, bone quantity, bone strength, implant position and inter-arch relationships are required to plan the proposed prosthesis design, implant surgery (including position, number and angulation) and adjunctive grafting procedures.

As with all treatment involving the use of dental implants, planning implants in the posterior maxilla should be restoratively driven. The design of the planned prosthesis should be ascertained first and all stages of prosthesis design, implant placement and tissue augmentation should be determined with the definitive prosthesis in mind. Most classifications relevant to implant planning in the posterior maxilla assess the severity of bone resorption, which in turn helps determine case complexity. However, the majority primarily focus on the surgical aspects of treatment, such as implant position, implant placement protocols and adjunctive bone augmentation. Very few include details of prosthodontic planning, and none focus primarily on restoratively driven planning.

The aim of this paper is to promote restoratively driven planning by describing the prosthodontic considerations to plan implants in the posterior maxilla, with a focus on augmented maxillary sinuses. It is not in the remit of this article to describe the surgical stages of maxillary sinus augmentation or implant placement in detail; therefore, further reading in these areas is encouraged.^2^ A new classification, the Posterior Maxilla Prosthodontic Index (PMPI), is proposed to promote restoratively driven planning for implants in the posterior maxilla.

## Prosthodontic planning for implants in the posterior maxilla

**Fig. 1:**
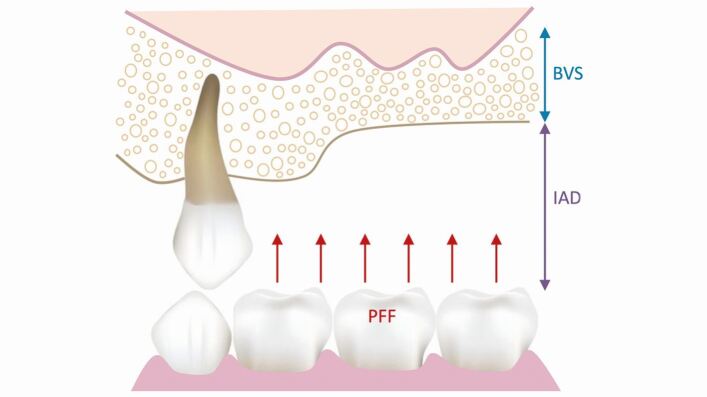
Diagram representing the intra-oral factors that need to be considered to plan prosthodontic options: BVS, IAD and PFF

**Fig. 2:**
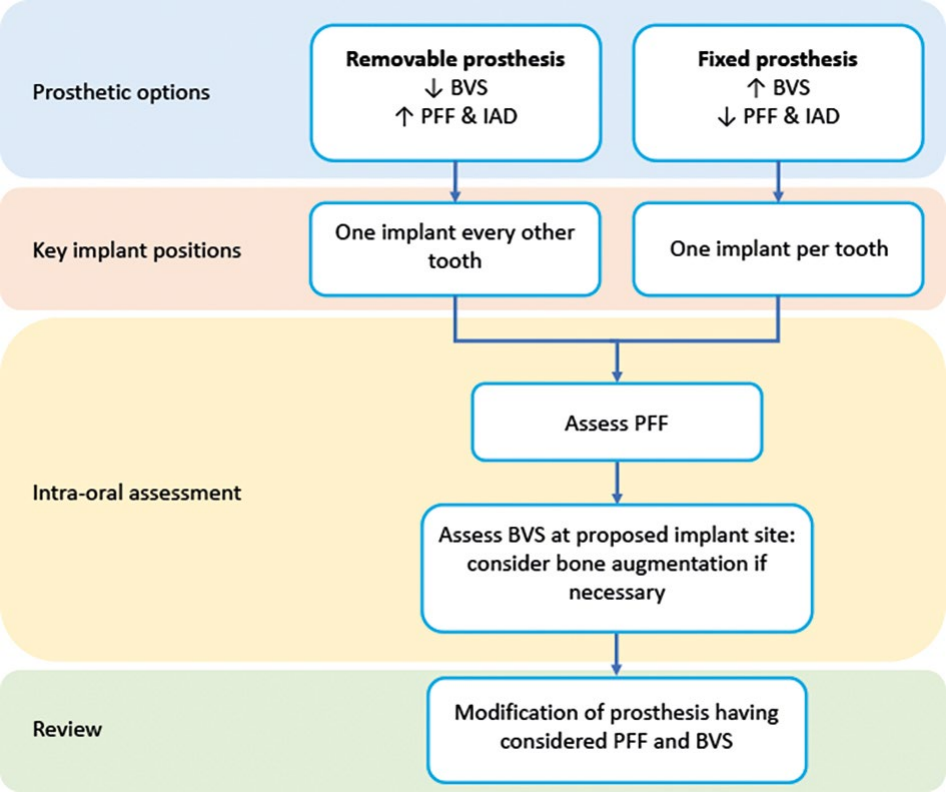
Treatment planning sequence for prosthodontic options when considering IAD, PFF and BVS

Planning restorations in the posterior maxilla requires comprehensive knowledge of how alveolar anatomy and interocclusal functional forces will influence prosthesis design, implant planning and any adjunctive surgical procedures. Surgical decisions, such as implant position, angulation and length, will influence the dissipation of occlusal forces through the prosthesis, implant and surrounding bone. Prosthetic factors, such as prosthesis height and cantilevered design, will affect the degree of non-axial loading and it is important to understand the associated biological and biomechanical consequences with such restorations. Augmentation procedures, such as maxillary sinus or alveolar ridge augmentation, can help overcome certain surgical and prosthodontic constraints to improve final prosthesis delivery and thus should be considered as part of restoratively driven planning. Following intra-oral bone assessment (Part 1),^1^ decisions for the implant rehabilitation of the posterior maxilla can begin. Figure 1 represents the intra-oral factors, namely bone volume and strength (BVS), inter-arch distance (IAD) and patient force factors (PFF), that need to be considered when planning prosthetic options. Figure 2 outlines the decision-making sequence that can aid the treatment planning for prosthetic options considering PFF and BVS. As the BVS decreases and IAD and PFF increase, the favoured implant-retained prosthesis is removable. As the BVS increases and IAD and PFF decrease, the implant-retained prosthesis can be either removable or fixed.

### Inter-arch relationships

Sinus grafting provides adequate bone height within the sinus to place dental implants; however, it does not correct compromised vertical or horizontal inter-arch relationships. It is essential to assess the IAD to establish whether the vertical height discrepancy can be accounted for in the prosthesis design, or whether vertical ridge augmentation is indicated. However, it must be stressed that the outcome of vertical ridge augmentation is unpredictable and therefore accounting for the vertical height defect through prosthetic design is often necessary.^3^ Likewise, it is important to assess the degree of horizontal bone loss and how this may impact treatment planning. As most resorption occurs buccally, the resulting maxillary ridge is often sited palatal to its original position. Thus, the ideal positioning of teeth and implants in relation to the opposing occlusion can be compromised. Impressions, accurately mounted study models and a diagnostic wax-up of the definitive tooth position should be performed at the earliest convenience to identify the pattern of alveolar resorption and identify suitable prosthetic or surgical treatment options.

### Occlusal force dissipation

Teeth are naturally shaped to dissipate occlusal forces axially through the tooth into the alveolar bone. Maxillary molars typically have three roots which extend up the buccal cortices of the maxillary alveolar process. The buccal cusp of the mandibular molars occludes with the central fossa of the maxillary molars, directing occlusal forces axially. Axial forces are dissipated favourably through the tooth, periodontal ligament and alveolar bone.^4^ However, an implant has significantly different anatomy to natural maxillary molar teeth. Implants are conical in shape, with the coronal portion positioned in cortical bone and the apical portion in highly cancellous and/or grafted bone. Furthermore, with the absence of a periodontal ligament, the implant-bone interface absorbs all the occlusal forces. Occlusal forces transfer from the prosthetic components to the implant, the implant-bone interface and finally, the surrounding bone, mostly at the crestal bone, with some dissipating at the middle and apical thirds.^5^ Depending on magnitude and direction of occlusal forces, it is possible for the maxillary native and/or grafted bone to become overloaded with insufficient prosthodontic and surgical planning, with the crestal bone being most at risk of pathological overload.

### Non-axial loading

Increased inter-arch distance following post-extraction resorption can typically be overcome through prosthesis design: vertical discrepancies are overcome using an increased crown-implant ratio (C:I) and horizontal discrepancies can be overcome using cantilevered restorations or tilted implants. However, the resulting prosthesis risks transferring non-axial forces through the implant into the surrounding bone. It has been theorised that non-axial occlusal forces have the potential to cause biologic and technical complications. This is particularly relevant in the posterior maxilla considering the less favourable bone anatomy and strength.

**Fig. 3:**
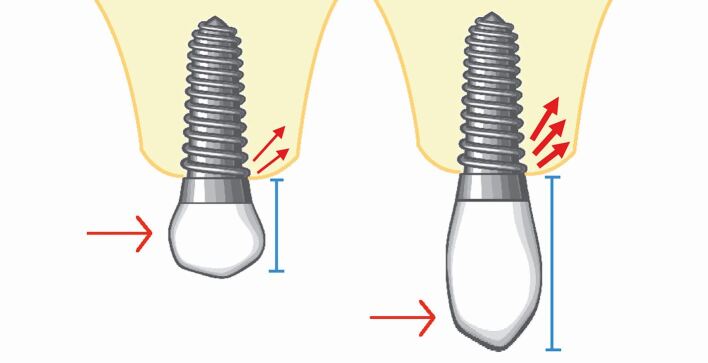
Diagram representing crestal forces comparing implants with favourable and unfavourable crown-implant ratios. Non-axial loading increases as the crown length increases. Long crowns act as a lever arm, creating bending moment and transference of occlusal forces to the peri-implant crestal bone

**Fig. 4:**
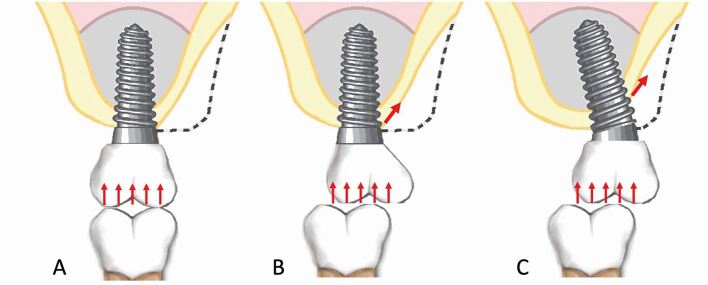
Diagram representing how tilted implants and buccally cantilevered restorations can create non-axial occlusal loading due to the position of the superstructure in relation to the implant fixture

The C:I of natural maxillary teeth has a mean value of 0.6.^6^ Traditional prosthodontic principles suggest a minimum C:I of 1:1 for abutment teeth.^7^ To avoid overloading of implants, these traditional prosthodontic principles have been applied to the C:I of implant-retained prostheses. The C:I defines the relationship between the length of the crown and the length of the implant. The C:I can be defined in two ways: 1) prosthodontically, whereby the crown-implant boundary is between the crown margin and the implant platform; and 2) clinically, whereby the crown-implant boundary is between the crown/abutment/implant collar and the level of the bone. For the purpose of this article, the clinically defined boundary is used to define C:I. Unfavourable C:Is cause non-axial loading as the longer crown acts as a lever arm, creating bending moment and transfer of occlusal forces to the peri-implant crestal bone (Fig. 3). High levels of stress during bending moments have been demonstrated around the necks and apices of implants, as well as along the implant body.^8^ A C:I equal to or less than 1:1 is considered ideal, with a C:I higher than 2:1 considered a high risk for biological complications.^9^ Tilted implants and buccally cantilevered restorations also create non-axial occlusal loading due to the position of the superstructure in relation to the implant fixture (Fig. 4).

### Biological complications

*In vitro* photoelastic models of implants in augmented maxillary sinuses show that when graft stiffness is lower than the native bone (immature graft), most occlusal stress is transferred to the native cortical bone and high levels of stress transfer to the immature graft.^10^^,^^11^ When the graft becomes a similar stiffness (mature graft) to the native bone, there is a more equitable stress distribution between native and grafted bone, therefore suggesting early loading could lead to overloading of the native and grafted bone.^10^^,^^11^ Furthermore, non-axial loading causes 11 times more stress in the surrounding bone compared to axial loading, and when the crown height is increased from 10 mm to 20 mm, forces can increase up to 200%.^12^ These photoelastic models suggest early loading and non-axial loading risk of overload of native and grafted bone. Unfortunately, due to a lack of clinical evidence, it is unknown whether these findings are accurate *in vivo*. However, systematic reviews have determined that unfavourable C:Is do not have any discernible negative clinical effects on marginal bone levels.^13^^,^^14^ Likewise, recent systematic reviews have suggested that peri-implant bone loss is not influenced by cantilever extensions of implant-supported prostheses or tilted implants.^15^^,^^16^ It is important to note that these studies have been conducted on short or tilted implants in native bone and further research is required to establish the clinical effects of non-axial loading for implants in augmented maxillary sinuses.

### Biomechanical complications

*In vitro *evidence suggests that increased crown height does not influence the stress distribution on implant screws during axial loading; however, during non-axial loading, crown heights over 12.5 mm caused statistically significant increase to the stress distribution and potential damage to implant screws.^17^ Furthermore, increased crown height has shown to negatively affect the resistance of internal implant connections to external occlusal forces and can lead to a reduction of performance and resistance to the connection system *in vitro.*^18^ There is currently no evidence to confirm whether these observations are true clinically for unfavourable C:Is, cantilevered restorations or tilted implants.^16^

Considering the limited evidence, it is prudent that fixed implant-retained prostheses are designed to reduce non-axial loading to protect native and grafted bone and prosthetic components. The design features listed in Table 1 should be considered for fixed implant restorations supported by implants in the posterior maxilla. It is important to state that the aesthetic appearance of longer crowns, pink porcelain or inharmonious gingival margins must be discussed with the patient before treatment.

**Table 1:** Treatment planning considerations to control non-axial loading for fixed prostheses on implants in the posterior maxilla

Prosthesis design factors	Patient factors
Reduced occlusal table Narrow occlusal table Reduced number of prosthetic teeth	Identify forces from opposing dentition: natural dentition > implant-retained prostheses > removable prosthesis
Low cusp height	Reduce/eliminate parafunctional habits
Light contacts in centric relation	Aim for an occlusal scheme whereby posterior teeth disclude during function (ideally canine-guided)
No occlusal contacts (working side or non-working side) in excursive movements	
Avoid cantilevers where possible (buccal, distal and mesial)	
Splinting of implants to distribute occlusal load	
Aim for a favourable crown-implant ratio	

## Implant planning in the posterior maxilla

The stages of restoratively driven planning, including impressions (analogue or digital), diagnostic tooth set-up (traditional or virtual) and cone beam computed tomography scanning with a radiographic guide *in situ* determines the appropriate implant position, number, length and diameter to support the planned prosthesis. In the posterior maxilla, certain additional factors are considered with regards to implant planning.

### Implant position

Ideally, implants should be planned in a straight configuration in relation to the prosthesis. Multiple implants should be placed parallel to one another. This ensures that the majority of occlusal load transfers down the long axis of the prosthesis and implants, thus reducing the risk of non-axial loading. Significantly increased microstrain has been detected around tilted straight and offset implant placement under axial and non-axial loads in implant-supported prostheses implants placed compared to a straight configuration *in vitro*.^19^ However, a recent systematic review showed no clinical difference between intentionally tilted versus straight dental implants in the medium- to long-term (>5 years).^20^ It is important to note that the maxillary tilted implants included were in native bone in the premolar region with the aim to avoid the maxillary sinus region. Thus, it is unknown if implants placed more posteriorly (in poorer-quality bone) or in augmented sinuses would have the same clinical outcome.

### Implant number

Currently, no studies have evaluated the optimum number of implants required to support fixed restorations replacing multiple teeth in relation to success and survival of implants in the posterior maxilla or augmented maxillary sinuses. Therefore, recommendations for the number of implants required are based on expert opinion and clinical experience. When it comes to planning such cases, the number of implants required to support multiple teeth is dependent on the bone strength and volume, which is often compromised in the posterior maxilla and augmented sinuses. Therefore, it is sensible to place a minimum of one implant per missing tooth for fixed implant-supported restorations when restoring short-span edentulous spaces (≤3 teeth).

### Implant length

Clinicians have anecdotally used the longest implant possible to ascribe to traditional prosthodontic principles of ideal C:Is. Although longer implants allow for a greater surface area of osseointegration, most forces applied to the implant body are concentrated in the crestal 7-9 mm of bone.^21^ Therefore, implant length beyond this measurement does not counteract the effect of a reduced C:I. Encouragingly, recent high-quality systematic reviews comparing short implants (≤8 mm) in native bone to longer implants in augmented sinuses report that short implants placed in the posterior maxilla have good short-term success and survival rates.^22^^,^^23^^,^^24^ However, further randomised controlled trials with larger patient samples and an observation period of more than three years are needed, as well as more detailed data regarding success and survival of the associated prosthetic components.

### Implant diameter

Occlusal forces applied to the implant body concentrate in the crestal 7-9 mm of bone, therefore increasing implant diameter is an effective method of increasing bone: implant contact in the crestal region.^21^ Wide diameter implants are recommended in the maxillary sinus region, with regular diameter implants acceptable (and ideally splinted if two or more are used). Narrow diameter implants are contraindicated in the maxillary sinus region due to the lower bone: implant contact and reduced strength of narrow implant components.

## Provisional prosthesis planning

It is important to consider provisionalisation in relation to implant success, particularly in relation to augmented maxillary sinuses. The aim is to eliminate movement of the implant and/or bone graft during healing, while providing the patient with acceptable function and aesthetics. For the first two weeks following implant or bone augmentation surgery, patients are advised against wearing a removable prosthesis to prevent pressure on the surgical site. Following the acute post-operative period, it is important to consider how implant loading affects the healing of the implant and augmented sinus before deciding upon a provisional prosthesis.

### Loading protocols

The literature regarding different loading protocols for implants in augmented maxillary sinuses is limited due to a lack of long-term data, absence of randomised controlled trials and limited studies with large numbers of patients. Histological evaluation of implants placed in autogenous and deproteinised bovine bone grafts following six months healing and subsequent loading show direct bone-implant contact and a similar bone density to native bone.^25^^,^^26^ Therefore, it is advised to allow 6-9 months of graft healing before functionally loading implants in augmented maxillary sinuses to allow for osseointegration and maturation of the grafted bone. It is now recognised that in certain clinical situations, controlled, immediate and early loading protocols do not interfere with implant osseointegration.^27^^,^^28^ For immediate or early implant loading, good primary stability must be achieved with an insertion torque values of >20-45 N/cm and implant stability quotient of >60-65 recommended.^29^ As posterior maxillary bone is often of the poorest strength, primary stability can be compromised and immediate implant loading is contraindicated. However, there has been increased reporting of the early loading of implants placed with simultaneous maxillary sinus augmentation.^30^^,^^31^^,^^32^ The results from two small clinical studies suggest that early loading of implants in the augmented maxilla does not affect implant survival. Caution is advised when extrapolating these results, as a very small number of patients were included and follow-up times were under one year.^30^^,^^31^ A higher-quality preliminary randomised controlled trial assessed implant survival in relation to the timing of sinus augmentation, implant placement and functional loading.^32^ The results showed that immediately restored implants, regardless of the timing of bone augmentation, had greater failure rates. The group with sinus augmentation at the time of implant placement with immediate loading was discontinued due to low insertion torque values and one implant failure. When traditional healing and loading protocols were followed (six months graft healing followed by six months implant healing), implant survival rates were the highest (100% at one year).^32^ Based on the current evidence, it is recommended that a graft consolidation period of six months, followed by an implant healing period of six months, is permitted before functionally loading implants placed in augmented sinuses for optimum success rates.

Progressive bone loading using modification of provisional prostheses over a period of time has been recommended in sites of poor bone density. Misch showed that bone matures when tension during the prosthetic phase increases gradually without overloading the implant.^33^ Progressively loaded bone reacts by increased formation, maturation and density, which can result in reduced crestal bone loss and early implant failure.^33^ Progressive bone loading can be considered for implants in augmented maxillary sinuses.

### Provisional restorations

Provisional restorations should be designed to minimise occlusal loads on the healing implant and consolidating graft. As already discussed, patients should avoid wearing a removable prosthesis for two weeks post-operatively to prevent impingement of the graft and soft tissue. Table 2 describes the advantages and disadvantages of the different provisional restorative options available following a two-week period of healing. Following implant exposure (after a minimum healing period of six months graft consolidation and six months implant integration), an implant-retained provisional restoration should be used if gingival contouring is required. Additionally, an implant-retained provisional can help assess function, aesthetics and the ability of the patient to maintain hygiene.

**Table 2:** Restorative options for provisional prostheses following implant placement in the posterior maxilla

Provisional type	Design features	Benefits	Risks
No provisional	-	No loading on implant/graft	Tooth tipping/rotating Over-eruption Poor aesthetics Poor function
Removable partial denture	Soft lining over graft - review and change regularly Good support, stability and retention - occlusal load spread through major connector utilising palate Minimise occlusal loads: Light occlusal contact in maximum intercuspation No occlusal contacts in mandibular excursions Shallow cusps Narrow occlusal table	Good aesthetics Provides function	Risk of loading implant/graft if designed inappropriately Removable prosthesis poorly tolerated in some patients
Prosthetic tooth in vacuum-formed retainer	Ensure coverage of all occlusal surfaces to prevent unwanted tooth movement	No loading on implant/graft Prevent unwanted tooth movement Moderate aesthetics	Poorer aesthetics than alternative options Poor function - needs to be removed during eating
Resin-bonded bridge	Metal or fibre reinforced composite framework Cantilever design Maximum surface area of retainer for bonding Occlusion: Light occlusal contact in maximum intercuspation No occlusal contacts in mandibular excursions	No loading on implant/graft Good aesthetics Provides function	Risk of de-bond Single tooth replacement only Requires unrestored/minimally restored abutment teeth

## Definitive prostheses planning

After a recommended minimum implant osseointegration period of four months in native bone or six months in grafted bone, a definitive restoration can be provided. Definitive restorations should be designed to reduce biomechanical load and prevent overload of the native and/or grafted bone. This can be achieved by considering the direction of load, occlusal design, cantilever length and splinting.

To optimise the dissipation of occlusal forces through the implant and bone, the aim should be to position implants in the ideal prosthodontic position in a straight configuration. A favourable C:I (<1:1) will reduce lever-arm forces and risk of crestal bone overload. The direction of occlusal load through the implants and surrounding bone is influenced by prosthodontic design. Avoidance of cantilevers will reduce the risk of non-axial loading. Cantilever lengths should be shortened or eliminated by planning a restoratively positioned implant, aiming to replace one implant per missing tooth. When multiple implants are placed to restore multiple teeth, splinting the implants via the restoration framework should be considered to control occlusal force distribution. Photoelastic analysis suggests that splinting implants improves stress distribution, improves retention and reduces stress on implant components.^34^ Careful occlusal design will also influence the intensity, direction and transfer of occlusal forces. It is recommended that initial light occlusal contacts occur on the natural dentition just before light contacts on the implant prosthesis in intercuspal position, with the idea that the periodontal ligament will absorb the majority of occlusal forces, thus minimising load on the implant(s). To prevent non-axial loading, no occlusal contacts should be present on implant-retained prostheses during excursive movements. Shallow cuspal inclinations and centrally oriented contacts will reduce lateral loads. A reduced occlusal table using narrower teeth or replacing fewer teeth (for example, one instead of two molars) will reduce occlusal load on the implant and graft.

Numerous *in vitro* studies have assessed material choice in relation to occlusal loading for implant-retained restorations. Acrylic resin and reinforced composite have been shown to transmit 25% and 15% less occlusal force than porcelain, respectively.^35^ However, the use of acrylic and composite as veneer materials does not seem to have a protective effect on the implant-bone interface.^36^ Regarding the framework material, alloys with a lower elastic modulus do not show substantial differences in stress patterns at the implant-bone interface of around the implant screw in finite element analysis.^37^ However, there are no clinical studies available to substantiate these results.

It is important to note that all restoration designs should prioritise cleansibility and minimal plaque retention for optimum long-term maintenance. The ability to brush and perform interproximal cleaning to the implant is essential to maintain the peri-implant tissues. By making all the mucosa-facing surfaces convex and polished, plaque retention will be minimised. All patients should be educated about how to clean around their implant-retained restoration.

## Restoratively driven clinical assessment

When restoring implants in the posterior maxilla, the prosthesis should be designed to reduce or mitigate non-axial forces on the prosthesis, implants, and native and grafted bone. Cases with increasing prosthodontic complexity increase the probability of non-axial loading, thus risking occlusal overload. To assess prosthodontic complexity, the prosthodontic envelope needs to be considered pre-operatively in a horizontal and vertical dimension using a diagnostic tooth set-up with the tooth/teeth in the ideal prosthodontic position. This enables planning of the appropriate prosthesis design, implant position and potential need for bone augmentation.

**Fig. 5:**
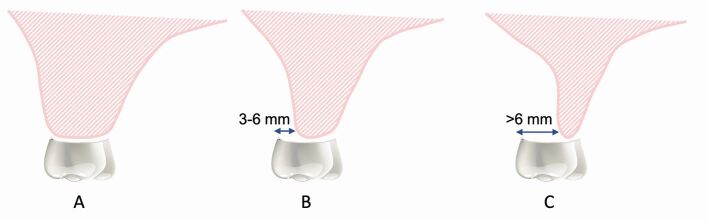
Diagram representing the CRP with the crown of the tooth in the ideal prosthodontic position. a) Crown in the ideal CRP with minimal (0-2 mm) horizonal resorption. b) CRP moderately compromised with the crown sitting buccal to the ridge with moderate (3-6 mm) horizontal resorption. c) CRP severely compromised with the crown sitting buccal to the ridge with severe (≥6 mm) horizontal resorption

**Fig. 6:**
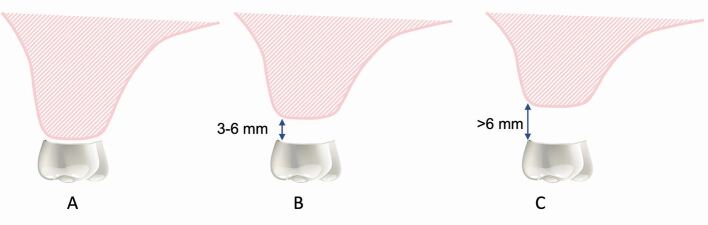
Diagram representing the IAD with the crown of the tooth in the ideal prosthodontic position. a) Cervical margin of crown close to ridge with optimal tooth height achievable (normal crown height +1-2 mm). b) Cervical margin of crown 3-6 mm from ridge with increased tooth/prosthesis height required (normal crown height +3-6 mm). c) Cervical margin of crown 6+ mm from ridge with increased tooth/prosthesis height required (normal crown height ≥6 mm)

**Fig. 7:**
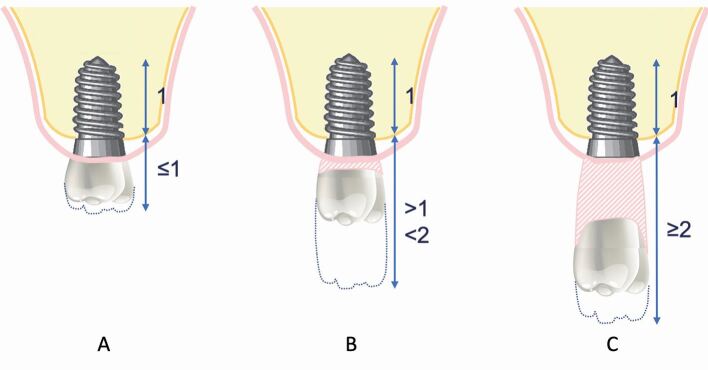
Diagram representing the C:I with the crown of the tooth in the ideal prosthodontic position. a) Ideal C:I where crown is equal to or smaller than the implant length. b) Moderately compromised C:I where crown is greater than the implant length but not more than double the implant length. c) Severely compromised C:I where crown is over double the implant length

To help establish the prosthodontic complexity of a case, the following factors should be assessed with the diagnostic try-in in situ: crown-ridge position (CRP), IAD and C:I. The CRP assesses the horizontal prosthodontic envelope and refers to the bucco-palatal position of the crown(s) on the diagnostic try-in to the edentulous ridge (Fig. 5). The IAD assesses the vertical prosthodontic envelope and refers to the space between the height of the edentulous alveolar ridge and the occlusion of the opposing arch (Fig. 6). The C:I defines the relationship between the length of the crown and the length of the implant (Fig. 7). The C:I can be defined in two ways: 1) prosthodontically, whereby the crown-implant boundary is between the crown margin and the implant platform; and 2) clinically, whereby the crown-implant boundary is between the crown/abutment/implant collar and the level of the bone. The biomechanical forces which transfer from an implant to crestal bone includes the crown, abutment and implant collar if using a tissue level implant. As previously mentioned, this article uses the clinical boundary to define the C:I. Assessing the CRP, IAD and C:I establishes the severity of alveolar resorption, as well as the prosthodontic and/or surgical treatment options to overcome mild, moderate and severe resorption.

### Horizontal assessment

The horizontal prosthodontic envelope can be assessed using the CRP with the crown(s) in the ideal prosthodontic position. With minimal buccal resorption, the crown(s) are centred over the ridge and the implant(s) can be placed in a straight configuration (Box 1). An optimal occlusal relationship can be achieved enabling axial loading of the prosthesis and implants. However, with increasing severities of buccal resorption, the crown(s) sit buccal to the resorbed ridge and alternative treatment options, such as a buccally cantilevered prosthesis, buccally angled implants, non-optimal occlusal relationships, or ridge augmentation procedures, are indicated.

Buccally cantilevered prostheses, angled implants or non-optimal occlusal schemes can overcome the need for adjunctive bone grafting procedures. Buccally cantilevered prostheses position the crowns in the ideal prosthodontic position, with the implant(s) being placed in a straight configuration in a palatal position (Box 2). With increasing severity of buccal resorption, angled implant configurations can be considered to achieve a fixed prosthesis without an excessive buccal cantilever and undercut. Angled implants move the implant platform buccally, while the apical end of the implant remains palatal (Box 3). However, both cantilevered prostheses and angled implants increase the risk of non-axial loading.^8^ Accepting non-optimal occlusal relationships (for example, cusp-cusp or posterior cross bite) can overcome the need for bone augmentation by accepting palatally positioned implants placed in a straight configuration and designing a prosthesis that corresponds with the long axis of the implant(s). This approach maintains axial loading; however, cross bites can result in reduced bite force and asymmetrical muscle function during chewing or clenching.^39^ To achieve the ideal implant position in relation to prosthesis design for horizontal ridge defects, ridge augmentation using guided bone regeneration or block grafting is indicated. If bone augmentation is contraindicated, a removable flanged prosthesis may be necessary.

Box 1 Case 1 **PMPI: simple horizontal, simple vertical**

- A 53-year-old woman attended for replacement of the 16 following loss of her tooth due to caries
- Horizontal and vertical assessment of the prosthetic envelope was performed using a diagnostic wax-up and tooth try-in
- CRP assessment identified horizontal resorption of less than 2 mm (minimal) was present allowing the crown to be centred on alveolar ridge in the buccolingual position
- IAD assessment identified vertical resorption of 1-2 mm (minimal) was present enabling the cervical margin of the crown to be situated close to the alveolar ridge thus enabling optimal tooth height (normal crown height +1-2 mm)
- The diagnostic wax-up and tooth try-in showed the ideal prosthodontic position was achievable with no ridge augmentation required
- Radiographic examination identified approximately 8 mm from the crest of ridge to the sinus floor. To achieve an optimal C:I of 1:1, sinus augmentation would be required
- A transcrestal sinus augmentation was performed at the time of implant placement to enable 10 mm implant to be placed to achieve an optimal C:I of 1:1
- A straight implant configuration was achieved with the crown in the ideal posterior occlusal relationship
- A crown of normal clinical crown height was placed.

Box 2 Case 2 **PMPI: moderate horizontal, simple vertical**

- A 30-year-old woman attended for replacement of the 26 and 27 following loss of tooth due to caries
- CRP assessment identified horizontal resorption of 3 mm (moderate) from the buccal aspect of the ridge was present
- IAD assessment identified less than 2 mm of vertical resorption was present (minimal)
- Radiographic examination revealed that there was less than 5 mm of alveolar ridge height present in the 26 and 27 region. Sinus augmentation would be required for implant placement and plan a favourable C:I of 1:1
- A lateral window sinus augmentation was performed, and two 10 mm implants were placed following a graft consolidation period of seven months
- A straight implant configuration was achieved. An ideal posterior occlusal relationship was achieved with buccally cantilevered single crowns
- Crowns of normal clinical crown height were provided.

Box 3 Case 3 **PMPI: complex horizontal, complex vertical**

- A 72-year-old woman attended requiring the restoration of implants placed in the left posterior maxilla. The implants had been placed by another dentist who was unable to restore them
- CRP assessment identified horizontal resorption of greater than 6 mm (severe) from the buccal aspect of the ridge
- IAD assessment identified greater than 6 mm of vertical resorption was present (severe)
- Radiographic examination revealed that a 12 mm (24), a 10 mm (25) and an 8 mm (26) implant were present
- The implants had been angled buccally to position the prosthesis in the ideal horizontal position. However, a buccally cantilevered prosthesis was still required to achieve the ideal posterior occlusal relationship
- Increased clinical crown height was required to overcome the increased IAD. Due to the good availability of vertical bone height, longer implants were placed, and C:Is of 0.75:1, 1:1 and 1.2:1 were achieved in the 24, 25 and 26 sites, respectively
- It must be noted that this treatment option significantly increased the complexity of prosthodontic delivery due to compromised access to the implant platform and the fabrication of custom angled abutments. The definitive restoration was cement-retained and a buccal cantilever of approximately 1-2 mm remained which risks oral hygiene problems and increased non-axial loading.

### Vertical assessment

Examining the IAD and C:I enables assessment of the vertical prosthodontic envelope. Assessing the IAD with the teeth in the ideal prosthodontic position determines whether the ideal crown height can be achieved, or whether there is an excess in prosthodontic space that needs to be corrected prosthodontically and/or surgically. The average crown heights for teeth in the posterior maxilla is listed in Table 3. With an increased IAD, space is present between the edentulous ridge and the cervical margin of the prosthodontic teeth (Box 1, Box 3, Box 4). The C:I is more complex to assess, as it requires knowledge of the length of the proposed implant(s), as well as the proposed prosthesis height. As previously described, alveolar bone height and sinus floor position will influence possible implant length; therefore, a comprehensive assessment of surgical (for example, sinus floor elevation) as well as prosthodontic parameters is required to determine the C:I. Assessing the C:I at the diagnostic try-in stage establishes the ideal implant length required to support the planned prosthesis. A C:I equal to or less than 1:1 is considered ideal (Box 1, Box 2, Box 3), whereas a C:I equal to or greater than 2:1 is considered a high risk for biological complications (Box 4).^38^ C:Is between 1:1 and 2:1 are considered unfavourable but have not been associated with biological complications (Box 3).^38^ It is currently not known what influence an increased C:I in the posterior maxilla has on prosthodontic complications.

**Table 3:** Average crown heights for posterior maxillary teeth^45^

Tooth	Average crown height
Maxillary first premolar	7.5 mm
Maxillary second premolar	6.5 mm
Maxillary first molar	5.5 mm
Maxillary second molar	5 mm

In cases with an increased IAD, prosthodontic treatment options for fixed prostheses include increasing the prosthesis height using longer clinical crowns or addition of pink porcelain, with the caveat that increased crown/prosthesis height will result in a higher C:I and risk of non-axial loading.^1^^,^^8^ In cases with significant vertical resorption, a larger IAD and a predicted C:I of 2:1 or greater, a removable flanged prosthesis is indicated to prevent implant overload and biological complications.^21^ To improve the C:I, sinus augmentation, or less predictably, vertical bone augmentation, is indicated to allow longer implants to be placed. It is important to consider that sinus augmentation alone will not correct vertical ridge defects, and an unfavourable C:I may still be present following sinus augmentation and the placement of longer implants if an increased IAD is present (Box 3 and Box 4). If sinus/ridge augmentation is contraindicated, shorter implants can be considered with the proviso that this will risk an unfavourable C:I.^40^

Once the horizontal and vertical prosthodontic envelope has been evaluated, additional prosthodontic considerations, such as screw access hole position and abutment selection, can be considered. The position of the screw access hole should ideally be mid-occlusal in the mesio-distal and bucco-palatal dimensions for maxillary posterior screw-retained prostheses. Accepting angled implant positioning changes the orientation of the access hole and increases the risk of emergence on the buccal aspect of the restoration. This can be an aesthetic issue, particularly in the premolar and first molar region. Alternatively, a cement-retained prosthesis can be considered; however, this increases the risk of biological complications and reduces ease of retrievability.^41^ Angled abutments of up to 45 degrees are available to help overcome angled implant positions; however, this again increases the risk of non-axial loading.

Box 4 Case 4 **PMPI: simple horizontal, moderate vertical**

- A 64-year-old man attended for replacement of the 26 and 27 following loss of tooth due to caries
- CRP assessment identified horizontal resorption of less than 2 mm (minimal) from the buccal aspect of the ridge
- IAD assessment identified 4-5 mm of vertical resorption was present (moderate)
- Radiographic examination revealed that there was less than 5 mm of alveolar ridge height present in the 26 and 27 region. Sinus augmentation would be required for implant placement and an unfavourable C:I would be accepted due to the increased IAD
- A lateral window sinus augmentation was performed and an 8 mm implant in the 26 and a 10 mm implant in the 27 sites were placed following a graft consolidation period of eight months
- A straight implant configuration was achieved with the crown in the ideal posterior occlusal relationship
- Crowns of increased clinical crown height were provided and a C:I of 2:1 was accepted. The crowns were splinted to improve the biomechanical loading on the implants and prosthetic components.

## Posterior Maxilla Prosthodontic Index

Due to the unique challenges associated with planning and providing implant-retained prostheses in the posterior maxilla, the PMPI has been developed to help initiate restoratively driven implant treatment planning. The PMPI is intended to be used at the diagnostic tooth try-in stage to assess the prosthodontic treatment options available to overcome implant-related restorative challenges in the posterior maxilla. Comprehensive alveolar bone assessment at the diagnostic tooth try-in stage helps identify whether unfavourable anatomical factors can be overcome through prosthesis design, or whether further surgical intervention (for example, ridge or sinus augmentation) is necessary. The PMPI is to be used alongside other aspects of clinical investigation, which should include clinical and radiographic assessment of the dentition, alveolar bone assessment and sinus floor position. The PMPI has been developed as an adjunctive tool to be used alongside other surgical planning indices for implant and bone augmentation in the posterior maxilla (for example, Misch and Judy; Jensen; Misch) so that prosthodontic and surgical treatment options can be systematically assessed at the outset of treatment.^42^^,^^43^^,^^44^ The full PMPI is available in Table 4.

**Table 4:** Posterior Maxillary Prosthodontic Index

	Clinical assessment			Treatment options				
	Horizontal	Vertical		Implant position	Occlusion	Prosthesis height	Prosthesis type	Surgical considerations
	CRP	IAD	Crown:implant ratio					
**Simple** Ideal prosthodontic position achievable with no/minimal ridge augmentation (+/- sinus augmentation)	Crown/s centred on ridge in ideal prosthodontic position	Cervical margin of crown close to ridge with optimal tooth height achievable (normal crown height +1-2 mm)	≤1:1 Crown is equal to or smaller than the implant length	Straight implant configuration achieves ideal prosthodontic position	Ideal posterior occlusal relationship** of maxillary:mandiblar dentition achievable	Normal clinical crown height (+/- 1 mm)	Favours fixed prosthesis	Consider sinus augmentation to improve implant length and positioning
	Minimal alveolar resorption (0-2 mm)*							
**Moderate** Ideal prosthodontic position only achievable with moderate compromises to prosthodontic outcome or/ alveolar ridge augmentation (+/- sinus augmentation)	Buccal aspect of crown 3-6 mm from buccal aspect of ridge	Cervical margin of crown 3-6 mm from ridge with increased tooth/prosthesis height required (normal crown height +3-6 mm)	>1:1 ≤2:1 Crown is greater than the implant length but not more than double the implant length	Straight or angled implant configuration (≤25^o^) achieves ideal prosthodontic position	Ideal posterior occlusal relationship** only achievable with buccally cantilevered prosthesis or/cusp-cusp relationship of maxillary:mandibular dentition in occlusal set-up with implant/s in straight configuration	Increased clinical crown height required (+2-4 mm)	Favours fixed prosthesis	Consider ridge or sinus augmentation to improve alveolar parameters, implant length and implant positioning
	Moderate alveolar resorption (3-6 mm)*							
**Complex** Ideal prosthodontic position only achievable with significant compromises to prosthodontic outcome or/ ridge augmentation (+/- sinus augmentation)	Buccal aspect of crown ≥6 mm from buccal aspect of ridge	Cervical margin of crown 6+ mm from ridge with increased tooth/prosthesis height required (normal crown height ≥6 mm)	>2:1 Crown is over double the implant length	Angled implant configuration (≤25^o^) cannot achieve ideal prosthodontic position	Ideal posterior occlusal relationship** only achievable with angled implants or/removable flanged prosthesis or/posterior cross bite^†^ in occlusal set up with implant/s in straight configuration	Significantly increased crown height (≥5 mm) requiring pink porcelain or removable flanged prosthesis	Favours removable prosthesis	Consider ridge or sinus augmentation to improve alveolar parameters, implant length and implant positioning
	Severe alveolar resorption (≥6 mm)*							
Key: * = Measurements based on the modified Siebert classification^46^ ** = Mandibular buccal cusps occlude along the central fossa of the maxillary dentition. Maxillary lingual cusps occlude along the central fossa of the mandibular dentition † = Maxillary buccal cusps occlude along the central fossa of the mandibular dentition								

### PMPI clinical application

A diagnostic tooth try-in with the prosthodontic teeth in the ideal prosthodontic position is required before using the PMPI. This can be achieved using traditional clinical try-in methods (for example, prosthetic teeth mounted in wax, removable partial denture, or vacuum-formed retainer) or using a virtual mock-up using digital planning software. With the diagnostic tooth try-in *in situ*, assessment of the horizontal and vertical prosthodontic envelope is undertaken using CRP, IAD and C:I.

**Fig. 8:**
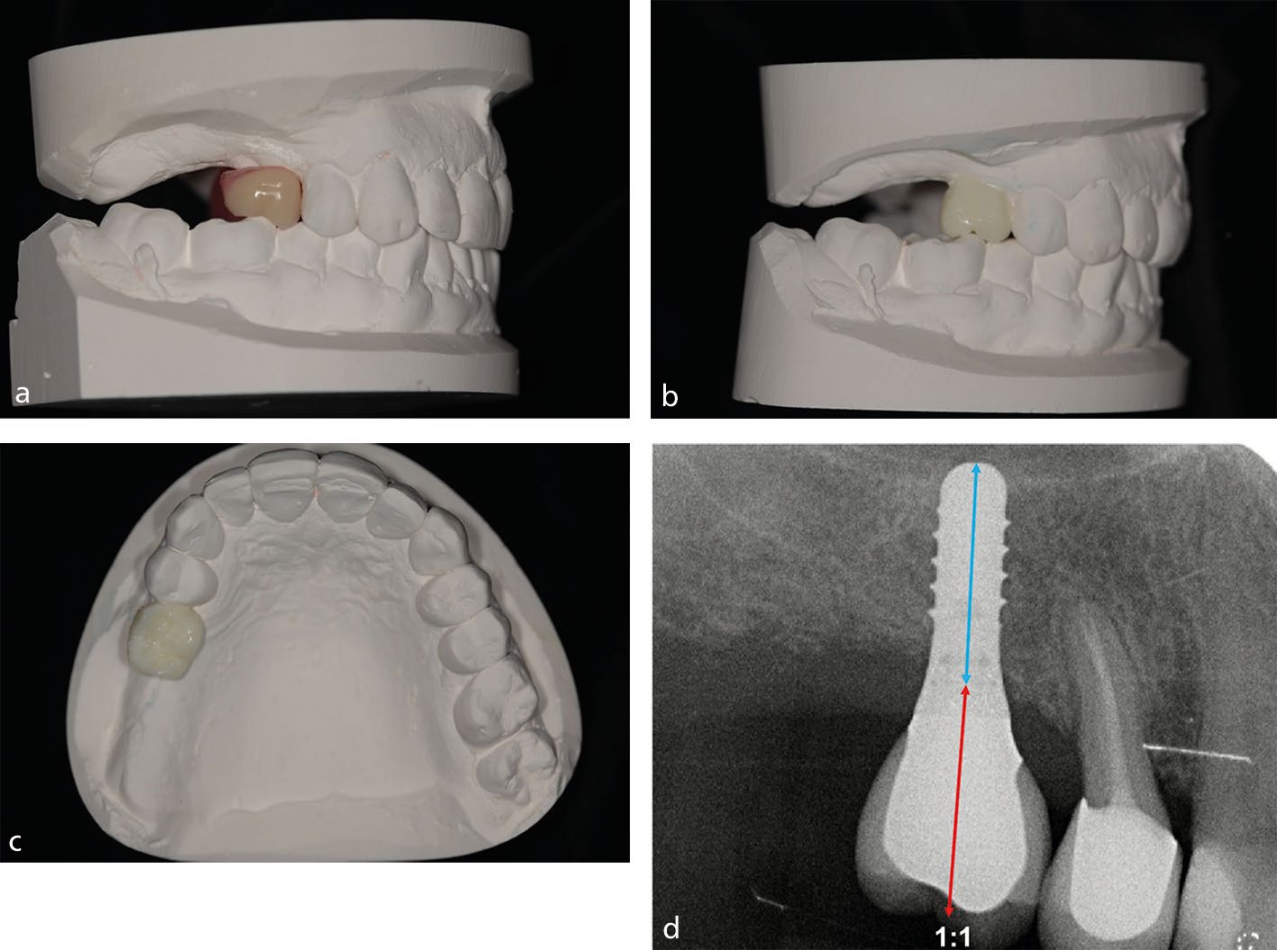
a) A diagnostic wax-up using a tooth of normal clinical height showing space between the height of the ridge and the cervical margin in the site of the 16 with occlusal contacts present in inter-cuspal position. This indicates that there is an increased IAD of approximately 1-2 mm. b) Modification of the wax-up from 16 with increased clinical crown height to accommodate for increased IAD. Estimated C:I can be determined using this wax-up as a guide. c) CRP showing crown centred on ridge in ideal prosthodontic position on dental cast. d) A 10 mm implant in the 16 site and optimal C:I of 1:1

**Fig. 9:**
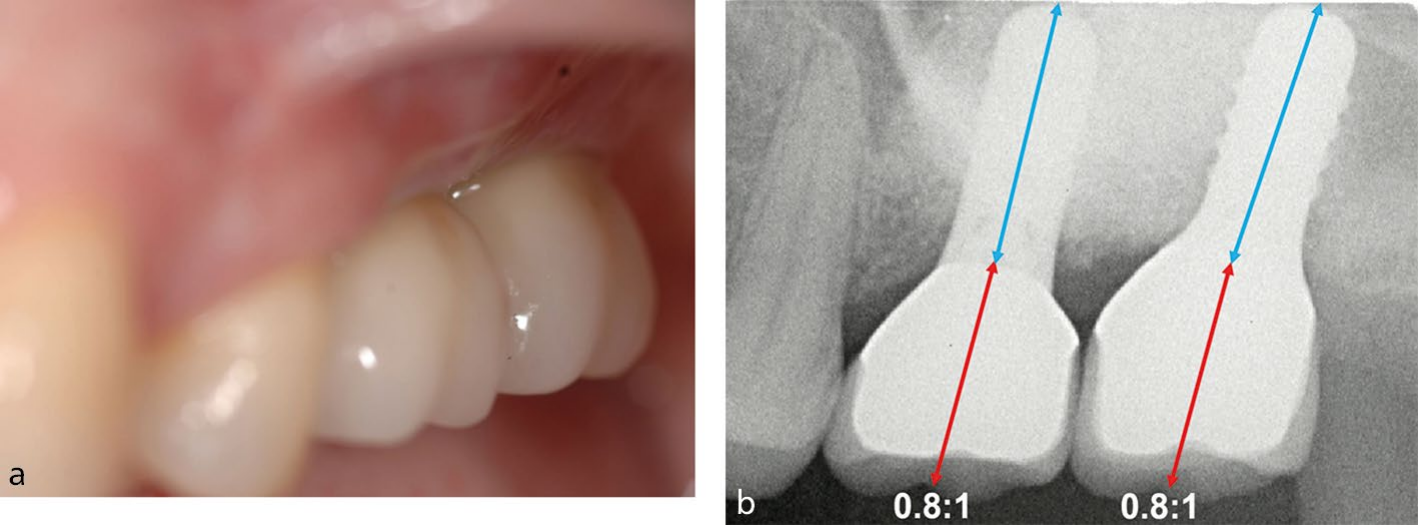
a) Buccally cantilevered crowns of a normal height used to achieve optimal occlusal contacts while allowing placement of implants in a straight configuration. b) Sinus augmentation enabled 10 mm length implants in the 26 and 27 regions enabling favourable C:I's of ≤1:1

**Fig. 10:**
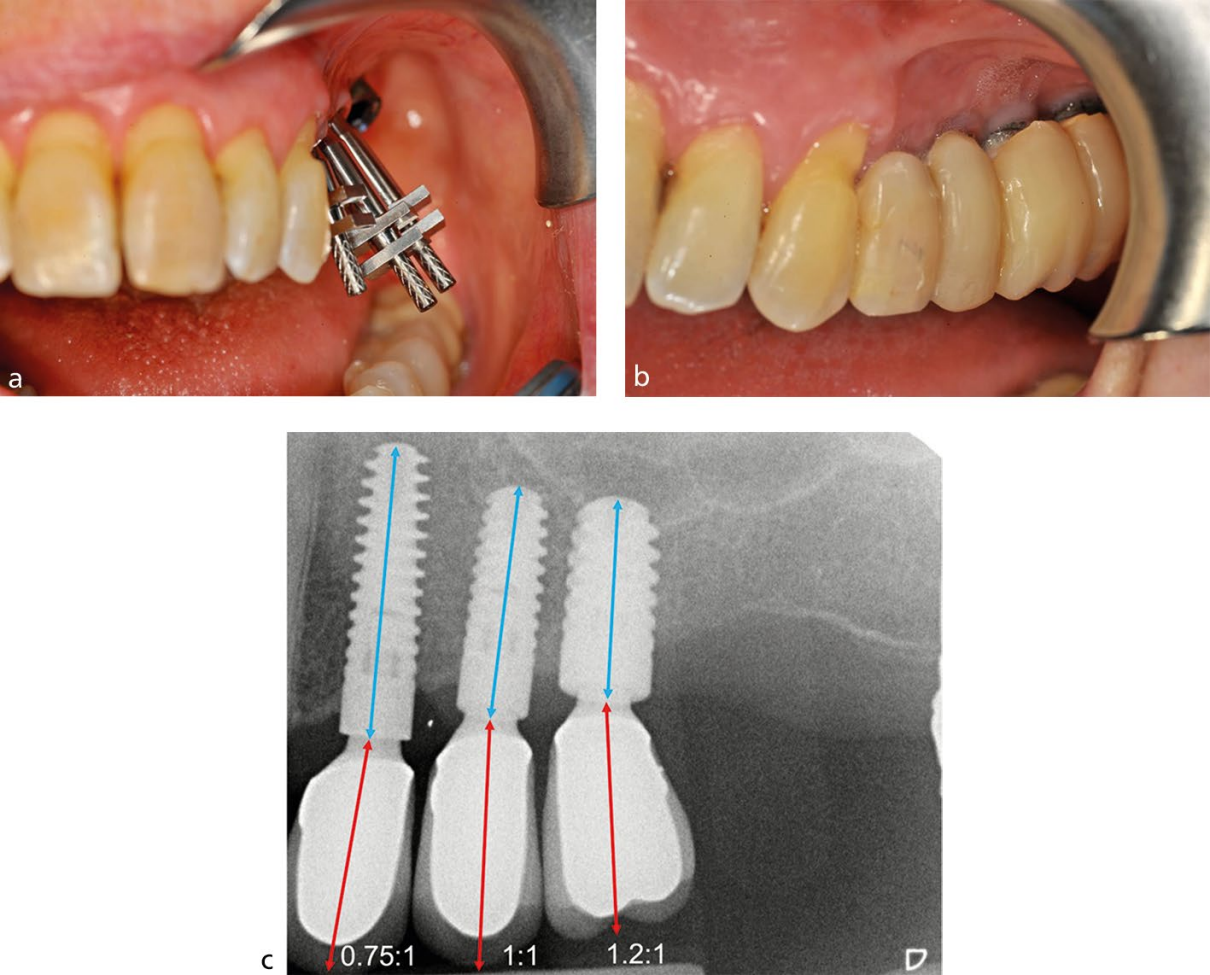
a) Buccally angled implants placed to overcome severe (6 mm) horizontal ridge resorption to achieve ideal crown position and ideal occlusal relationships. b) Try-in of temporary prosthesis. Note the compromised CRP and presence of buccal cantilever to achieve the ideal posterior occlusal relationship. c) IOPA radiograph showing ideal C:Is of ≤1:1 (24, 25) and moderately compromised C:I between 1:1 and 2:1 (26)

**Fig. 11:**
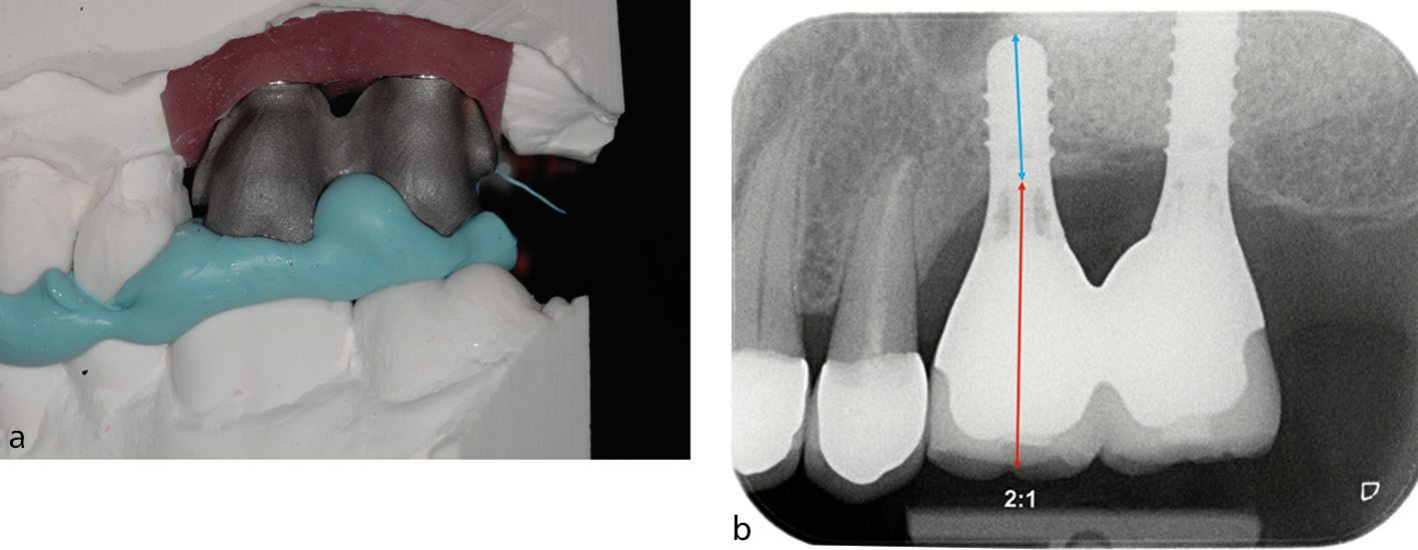
a) Metal substructure for splinted crowns restoring the 26 and 27 implants demonstrating 4-5 mm of vertical resorption (moderate) on dental cast. Minimal horizontal resorption thus straight implant configuration was achieved with the crown in the ideal posterior occlusal relationship without the need for a buccal cantilever. b) IOPA radiograph showing C:I of equal to or greater than 2:1 (26). Augmentation of the left maxillary sinus has enabled longer implant placement; however, increased C:I was still present due to increased IAD

Using the PMPI, a case can be categorised as 'simple', 'moderate' or 'complex' in both the horizontal and vertical dimensions, which identifies the complexity of prosthodontic rehabilitation. Clinical examples are demonstrated in Box 1 (Fig. 8), Box 2 (Fig. 9), Box 3 (Fig. 10) and Box 4 (Fig. 11). The PMPI provides prosthodontic treatment options to overcome posterior maxillary defects without bone augmentation, as well as surgical considerations. As previously discussed, prosthodontic planning decisions, such as crown height, implant angulation, C:I and cantilevered prostheses, influence the degree of non-axial loading on the implant, native alveolar bone and grafted bone.^1^ Therefore, the increasing PMPI risk categories are associated with an increased risk of non-axial loading and/or overload of the prosthesis, implant or bone. For moderate and complex cases, bone augmentation (for example, ridge augmentation and sinus augmentation) can be considered to improve the final prosthodontic envelope (CRP, crown height space and C:I). However, it is important to remember that implants in grafted bone are at a higher risk of biomechanical overload (see Part 1),^1^ thus prosthodontic design should always aim to reduce non-axial loading.

Figures 8, 9, 10 and 11 detail how the PMPI has been applied to different cases of dental implant rehabilitation in the posterior maxilla.

## Conclusions

Alveolar ridge anatomy directly effects prosthodontic delivery, as well as surgical treatment options. It is important that alveolar bone volume, inter-arch relationships and anticipated non-axial loading is assessed at the planning stage, as this will enable restoratively led implant planning. Assessing these parameters will determine the requirements of the definitive prostheses and thus establish the surgical aspects of treatment, such as implant number, angulation, diameter and length. Clinical assessment using CRP, IAD and C:I can help identify the severity of ridge defects, as well as establish the complexity of prosthesis or implant delivery. Assessing these clinical factors can help establish whether resorption/pneumatisation can be overcome with prosthesis design or ridge/sinus augmentation.

Appropriate restoratively driven planning helps identify and limit the risk of biological and prosthodontic complications and therefore helps to formulate informed consent, monitoring and maintenance requirements. The PMPI combines the assessment of CRP, IAD and C:I to classify the complexity of prosthodontic and implant treatment in the posterior maxilla. Furthermore, prosthodontic and surgical treatment options are suggested for the different complexities to encourage restoratively driven planning.
